# New Advertisements

**Published:** 1885-01-20

**Authors:** 


### NEW ADVERTISEMENTS.

The following new advertisements appear in this number of the RECORD, and will be found interesting, to wit: John C. Baker & Co.; Fairchild Brothers & Foster, and Dolliber & Goodale. Notice, also, changes of advertisement by Messrs. Parke, Davis & Co., of Detroit; Battle & Co., of St. Louis, Mo.; Landrum & Litchfield; Rio Chemical Company; National Pharmacal Association; New York Pharmical Association, etc.

---

